# Supplementary material for: TtCel7A: A Native Thermophilic Bifunctional Cellulose/Xylanase Exogluclanase from the Thermophilic Biomass-Degrading Fungus Thielavia terrestris Co3Bag1, and Its Application in Enzymatic Hydrolysis of Agroindustrial Derivatives
Source: J Fungi (Basel). 2023 Jan 23;9(2):152. doi: 10.3390/jof9020152 (PMC9961574; doi:10.3390/jof9020152)
Supplement: Supplementary file 1 [file jof-09-00152-s001.zip › jof-2142636-supplementary.pdf]

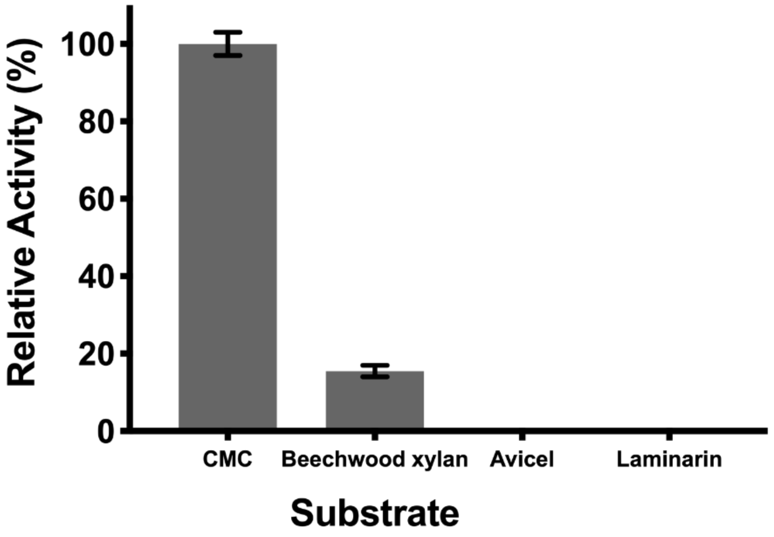

Figure.S1. Effect of substrate on the activity of purified TtCel7A. The activity was assayed using CMC, **Beechwood** xylan, Avicel, or Laminarin at a final concentration of 0.6 % (w/v) in 50 mM acetate buffer, pH 5.5 at 60 ºC.

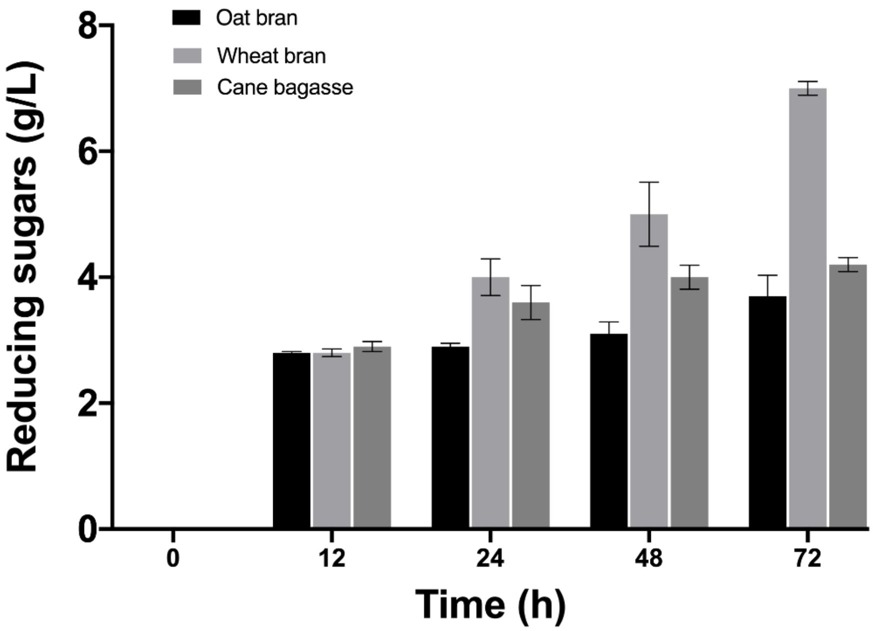

Figure. S2. Reducing sugars released from Oat bran, Wheat bran, or Cane bagasse after incubation with purified TtCel7A (30 mg/mL).

MHAKFAT**LAALVA**SAAA**QQACTLTAENHPTLSWSKCTSGGSCTSVSG**A**VTIDANWR**WTHQVSSSTNCYTG
NEWDTSICTDGASCAAACCLDGADYSGTYGITTSGNALSLQFVTQGPYSTNIGSRTYLMASDTQYQTFTL
LGNEFTFDVDVSGLGCGLNGALYFVSMDADGGLSKYSGNK**AGAKYGTGYCDSQCPR**DLKFI**NGEANNVGW**
TPSSNDKNAGLGNYGSCCSEMDVWEANSISAAYTPHPCTTIGQTRCEGDACGGTYSTDR**YAGECDPDGCD**
FNSYRMGNTTFYGKGLTVDTSK**KFTVVTQFLTDSSGNLSEIKRFYVQNGVVIPNSNSNIAGVSGNSITQA**
FCDAQKTAFGDTNVFDQKGGLAQMGKALAQPMVLVMSLWDDHAVNMLWLDSTYPTDAAGKPGAAR**GTCPT**
TSGVPADVESQAPNSKVIYSNIRFGPIGSTVSGLPGGGSNPGGGSSSTTTRTSTTTRTTTSSSAGTSPTG
GTAPHWGQCGGIGWTGPTVCASPYTCQK**LNDWYYQCL**

Figure. S3. Amino acid sequence of TtCel7A from *T. terrestris* Co3Bag1 (Accession number: AQM74408). Peptides obtained by amino acid partial sequencing of purified TtCel7A by MS/MS are highlighted in blue. The amino acid sequence used for primer design are underline.

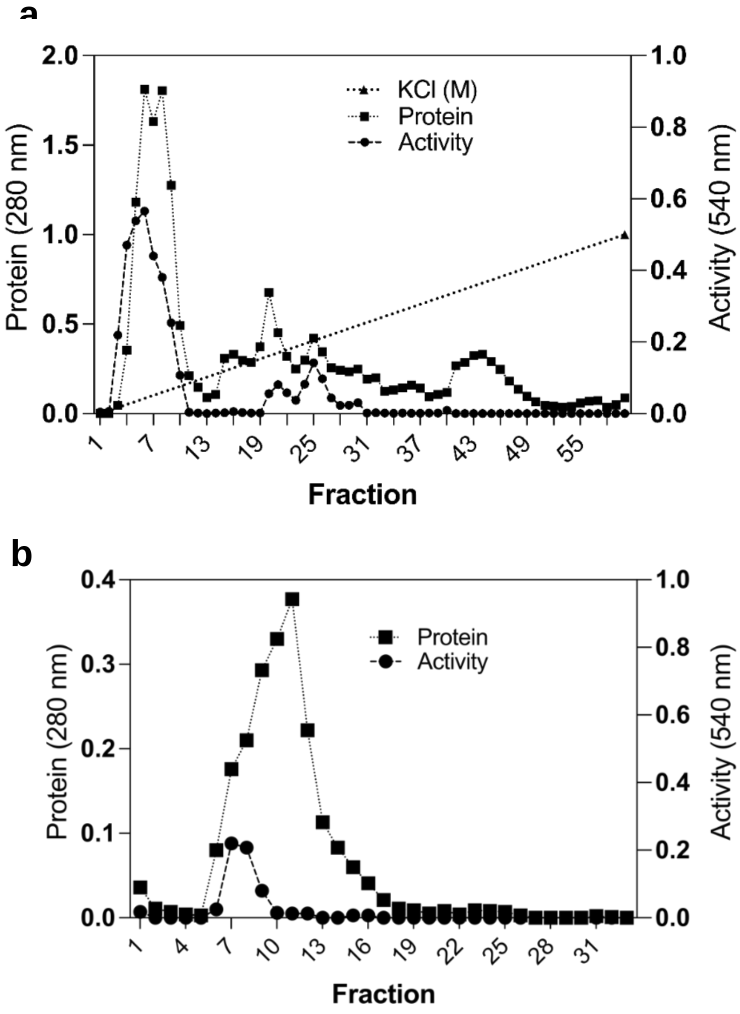

Figure. S4. (**a**) Anion Exchange Chromatography of Cel7A purification. A gradient of 0.0 to 1M of KCl was used for protein elution. Fractions between 19 and 31 section were pooled for the next purification step. (**b**) Gel filtration Chromatography purification, 50 mM acetate buffer, pH 5.5 was used for protein elution, fractions with enzymatic activity were used for further studies. (■) protein 280 nm, (●) enzyme activity 540 nm, (▲) KCl concentration.

Table S1. Primers used for DNA sequencing of Ttcel7A gene

**Primer 1:** 5´-GACCCTCGCCGCCCTTGTGG-3’
**Primer 2:** 5´-SRCYCTCGCCGCCCTYGTGG-3
**Primer 3:** 5’-TGCGACCCTGACGGATGCGAC-3’
**Primer 4:** 5´-TGCGACCCYGAYGGMTGCGAC-3´
**Primer 5:** 5’-GAGGCACTGGTAGTACCAGTC-3’
